# Pivoting to online laboratories due to COVID‐19 using the Science of Agriculture digital tools: A case study

**DOI:** 10.1002/nse2.20045

**Published:** 2021-04-28

**Authors:** Eric C. Brevik, April Ulery, Amy Smith Muise

**Affiliations:** ^1^ Dep. of Natural Sciences and Dep. of Agriculture and Technical Studies Dickinson State Univ. Dickinson ND 58601 USA; ^2^ Dep. of Plant & Environmental Sciences New Mexico State Univ. Las Cruces NM 88003 USA; ^3^ Dep. of Innovative Media Research & Extension New Mexico State Univ. Las Cruces NM 88003 USA

## Abstract

On 12 Mar. 2020 Dickinson State University moved all classes to distance delivery (DD) in response to COVID‐19. Faculty had only a brief opportunity to plan, as the turn‐around to DD was very rapid. Meaningful laboratory exercises were needed for SOIL 210–Introduction to Soil Science. The senior author learned about the Science of Agriculture (SoA) website on a discussion board created by the Soil Science Society of America, which provided links to distance education resources. Most of the resources from SoA addressed topics still to be covered in SOIL 210, and four of the semester's final six labs were developed using SoA: Understanding Data and Chemistry, Soil Chemistry, Dryland Soils, and Microbiology and Nitrogen. Materials available on the SoA website include video clips, interactive exercises, and virtual labs. Although the virtual labs, with the exception of Sorption!, are not soil science focused, they cover basic skills that soil scientists use. Each of the four labs utilized four to eight of the activities (video clips, interactive activities, and/or virtual labs) available on SoA, depending on the length of time each activity was expected to take and the number of activities available for the given topic. Students were asked to answer specific questions related to their lab experience with the digital activities. The SoA website provided useful tools to develop meaningful experiences for the SOIL 210 students in lieu of their traditional laboratory exercises.

**Abbreviations**
DSU: Dickinson State University
NMSU: New Mexico State University
SoA: Science of Agriculture website
SOIL 210: Introduction to Soil Science
UWS: Understanding Western Soils website
VL: Virtual Labs website.

## 1 INTRODUCTION

Spring semester 2020 brought about a nearly universal shift to distance delivery of coursework in the global higher education system (Fox et al., 2020). The American Geosciences Institute found that all geoscience departments that responded to their survey on delivery method shifted from face‐to‐face to online‐only instruction during the spring 2020 semester (Gonzales & Keane, 2020). Dickinson State University (DSU; Dickinson, ND, USA) made the decision to move to distance delivery of all coursework on 12 Mar. 2020. The following week was spring break, which gave faculty a short amount of time (9 days including the weekends on each end of spring break) to prepare for the shift. However, this was much more rapid than ideal, leading some to argue that a term such as “emergency remote teaching” should be used to describe what happened during the spring of 2020 rather than “online learning” (Hodges et al., 2020). The sudden shift caused considerable stress among both faculty and students (Gibbs, 2020), in no small part because many of these faculty and students had no previous experience teaching or learning at a distance and were ill‐prepared to participate in it (Means et al., 2020). This was much the circumstance the senior author found himself facing in March 2020. Although pivoting to distance delivery of lectures can be achieved with relative ease—given the availability of online platforms like Blackboard Collaborate, Zoom, and Google Meet—pivoting in‐person, hands‐on learning that occurs in lab settings presented more of a challenge.

## 2 THE CASE

### 2.1 Teaching notes

One of the classes taught by the senior author during spring 2020 was SOIL 210–Introduction to Soil Science (SOIL 210). This four‐semester‐credit class includes three 50‐minute lecture periods and one 110‐minute laboratory period per week. Class enrollment typically ranges from 20 to 45. The class is taught once per year with two laboratory sections. Programs at DSU that use SOIL 210 as part of their curriculum include the associate of science (AS) in agricultural sales and services (Agricultural Business Management, Natural Resource, and Production Agriculture options); the Bachelor of Science (BS) in agricultural studies (all options); BS in biology (Organismal Biology option); BS in environmental science; Farm and Ranch Management certificate; and the soils minor. This class and laboratory have always been taught face‐to‐face, with no serious consideration given to an online offering of the class prior to March 2020. Therefore, when DSU went completely online there were no previous plans in place for online teaching of, most particularly, the laboratory materials.

The fundamental skills that students are expected to gain in the SOIL 210 laboratory during a typical semester are:

1. Identifying soil parent materials and being able to discuss how parent materials influence the properties of soils
2. Utilizing the web soil survey (https://websoilsurvey.sc.egov.usda.gov/App/HomePage.htm) to determine the soils expected at a given location and to investigate their properties
3. Using the web soil survey as a tool in land use planning
4. Describing soil color, texture, structure, and other physical properties
5. Measuring bulk density, soil water content, pH, and soil organic matter
6. Being able to estimate annual water erosion in a field using the universal soil loss equation
7. Understanding the fundamental relationships between soils and landscapes.

**Core Ideas**

- COVID‐19 led to a rapid shift to online teaching during spring 2020.
- Lab classes provided a particular challenge for conversion to distance delivery.
- Animated videos, interactive activities, and virtual labs offered a way to fill the gap.
- The “Science of Agriculture” activities have been shown to improve student learning.
- Skills covered aligned well with topics covered in the second half of the semester.

**FIGURE 1:**
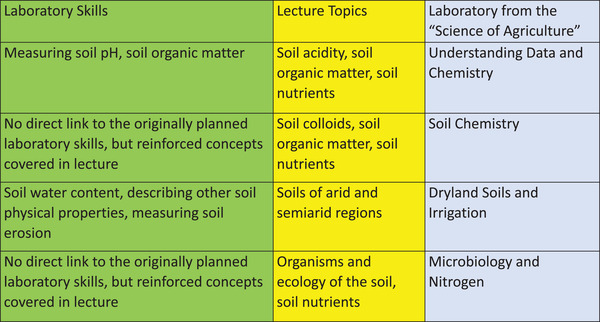
Links between the laboratory skills that were planned at the beginning of the semester, the lecture topics remaining when the class moved to distance delivery, and the laboratories that were developed using the Science of Agriculture website

At the time that classes moved to distance delivery in mid‐March, the first four skills had been addressed, whereas the final three had not. Six additional face‐to‐face laboratories had been planned to cover those three skill groups between the time of conversion to distance delivery and the end of the semester; therefore, there was a need to develop six meaningful online laboratories. In the lecture, topics yet to be covered at the time of conversion to distance delivery were (a) soil colloids, (b) soil acidity, (c) soils of arid and semi‐arid regions, (d) organisms and ecology of the soil, (e) soil organic matter, and (f) soil nutrients. Note that not all of the laboratory skills that were originally planned to be covered at the beginning of the semester were covered with the online lab activities (Figure 1). This issue is covered in more detail in the Discussion section.

## 3 THE DECISION

Several resources were investigated in an effort to find something that would fit the specific needs of SOIL 210 through the remainder of the spring semester. The Science of Agriculture (SoA; https://www.scienceofagriculture.org) collection offered a variety of online resources that provided educational content to the students that was relevant to topics still to be covered in class and presented in an engaging way. Several of the SoA activities were well aligned with the lecture topics remaining in SOIL 210 at the time of the shift to distance learning (Figure 1). Another important consideration was that previous research indicated that the SoA activities were successful in helping students grasp difficult soil science concepts (Ulery et al., 2020). Therefore, SoA was chosen for the development of several online laboratory activities for the second half of the semester.

The SoA collection includes educational tools that encourage students to engage with topics in various ways. Animated video clips explain important soil concepts with easy‐to‐follow graphics. Interactive activities allow students to have an active role in progressing the lessons through to their conclusions. The video clips are a few minutes each, typically lasting 4–8 minutes each. The interactive activities generally take 15–20 minutes and center on a number of soil and water issues that are important to an introductory level soil science student. There are also video clips and interactive activities that focus on building core skills important to a soil scientist (or scientists in general for that matter), such as graph reading and understanding logarithms. This content was developed via a research‐based method that incorporated formative testing and user feedback (Chamberlin et al., 2016). Topics were chosen based on findings that students engage well with multimedia animations conveying science topics of intermediate complexity (Ulery et al., 2020). The site also provides links to related collections of additional resources: Understanding Western Soils (UWS; https://westernsoil.nmsu.edu) and Virtual Labs (VL; https://virtuallabs.nmsu.edu). The VL exercises are not soil science specific, but several of them center on chemical and microbiological procedures that soil scientists use, and these were therefore considered appropriate for use in the SOIL 210 laboratory. The SoA, UWS, and VL collections are completely open sources, with no fees charged for their use and no registration or login process required. All three collections were developed by the New Mexico State University (NMSU) Department of Innovative Media Research & Extension in collaboration with agricultural scientists at NMSU and other institutions.

**FIGURE 2:**
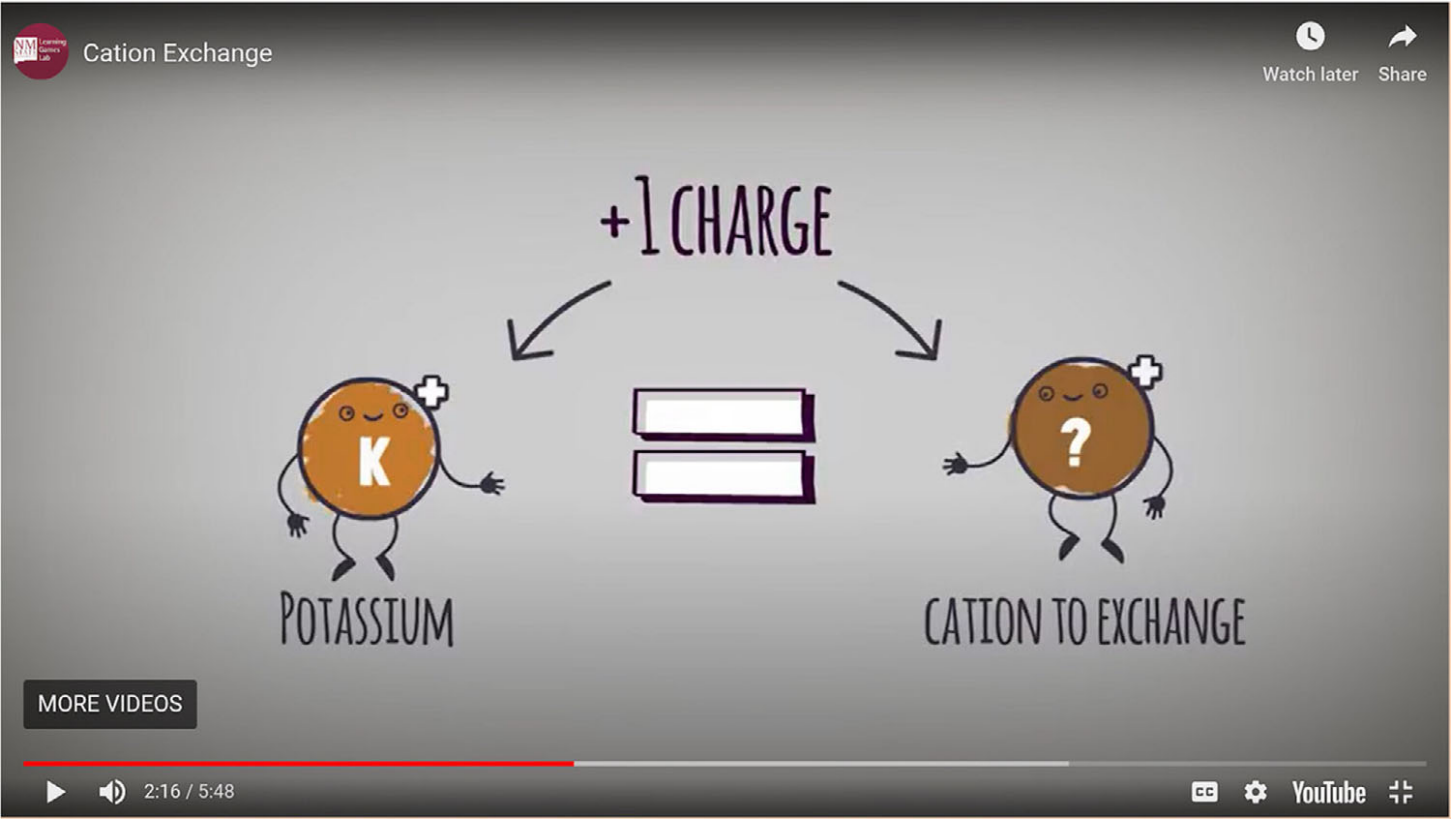
A screen capture from the animated video “Cation Exchange.” At this point in the video the students were asked, “What does a plant need to do if it wants a nutritious cation like potassium?” The animated explanation provided by the video has been shown to help students understand the cation exchange concept (Ulery et al., 2020)

**FIGURE 3:**
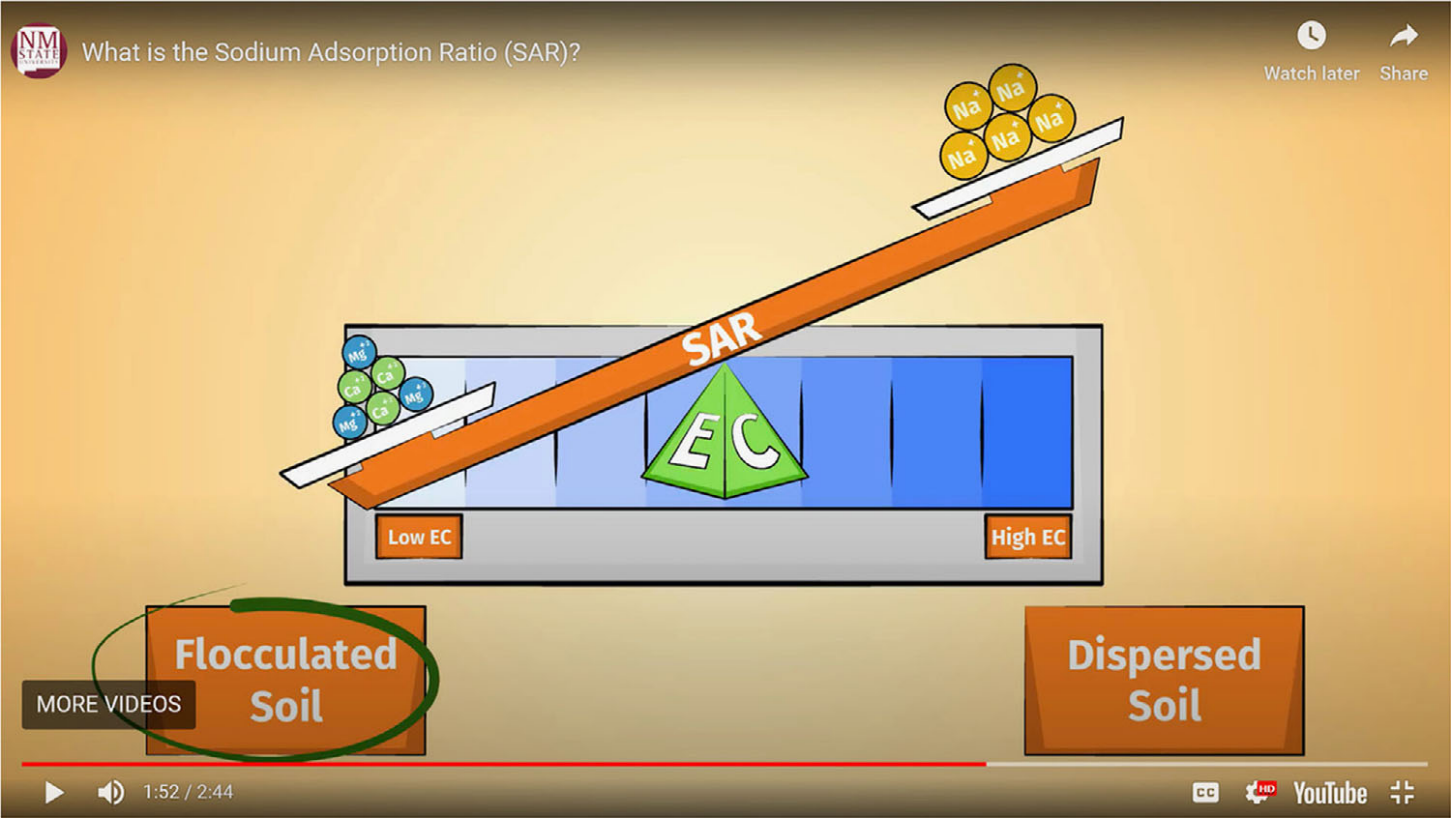
A screen capture from the “Understanding the Sodium Adsorption Ratio (SAR)” animated video. At about this point in the video the students are asked the question, “Does soil disperse or flocculate at high SAR values?” The question is not directly answered by the image above, but if the students have paid attention to the video they can answer the question with the information provided

**FIGURE 4:**
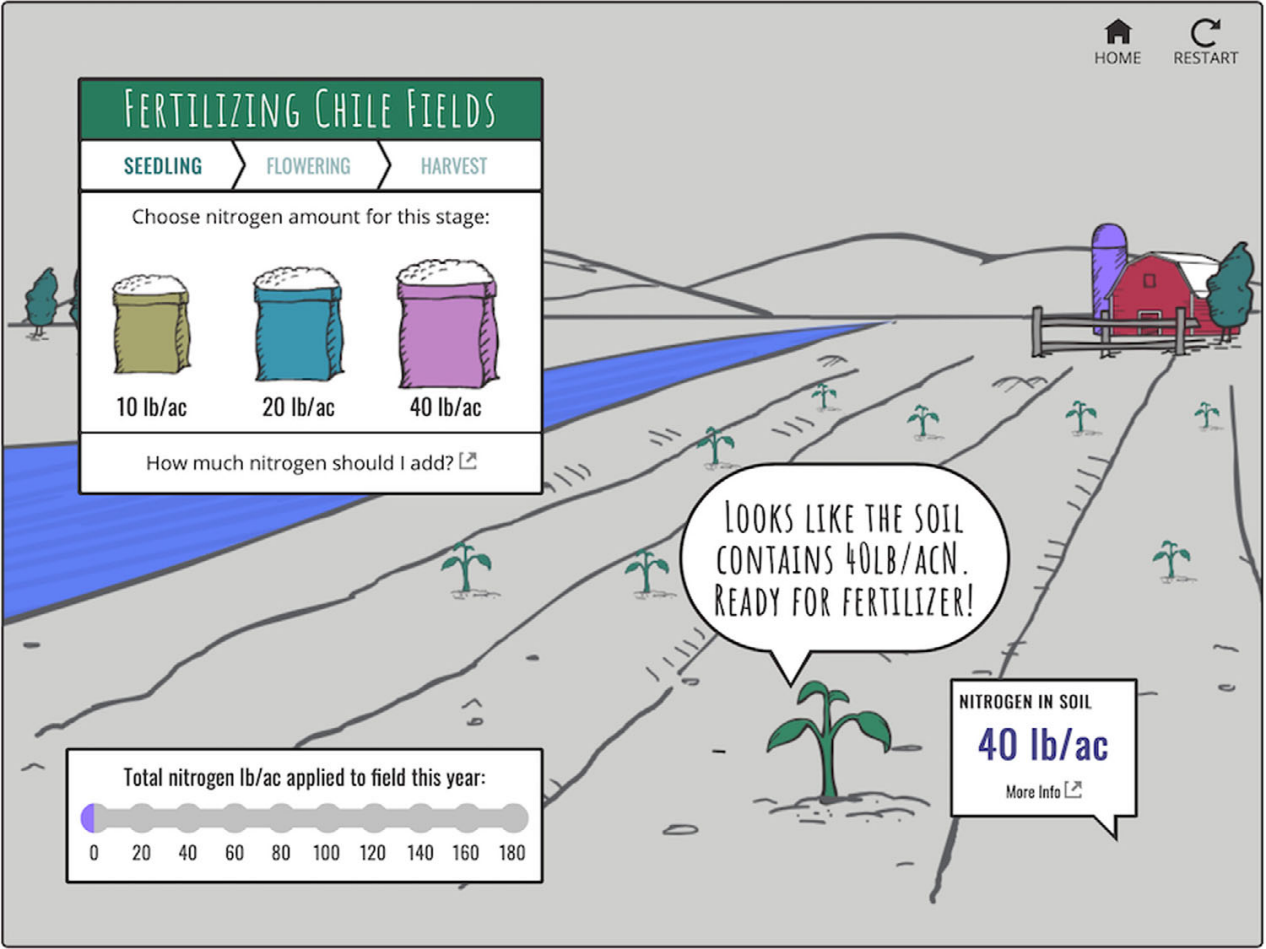
A screen capture from the “Nutritious Nitrogen” interactive activity. In this activity, students get a soil test and then need to choose how much nitrogen fertilizer to add at the seedling and flowering stages of crop development. At this point in the laboratory there are a series of questions that ask the students what happens if they add 10 lb/ac (answer: not enough nitrogen to grow or flower, so the interactive tells the student to try again) or 40 lb/ac (answer: too much nitrogen increases plant vegetative growth but prevents flowering; again, the student is told to try again). The student has similar fertilization choices at the flowering stage. At the end of the interactive activity, the students are asked a reflective question about why it is important to use appropriate nitrogen fertilizer amounts (not too much, not too little) and why soil nutrient testing is important. The interactive activity allows the students to “raise” plants as they might in a greenhouse on campus during a semester‐long experiment, but much more rapidly and from a remote (socially distanced) location

Four of the final six laboratories for the spring 2020 semester were developed from the SoA, UWS, and VL collections. A summary of the laboratories and activities used from each website is given in Table 1. The number of online activities utilized for individual laboratories ranged from four to eight (with an average of 6.25 per laboratory). The different numbers of activities used were due to different amounts of time needed to complete certain activities and the number of activities available for a given topic. Ideally each online laboratory would take the students, on average, about 2 hours to complete, and they were structured with that general goal in mind, but no data were gathered concerning how long they actually took. Several questions were asked of students as they worked through each video, interactive activity, or virtual lab. Screenshots of points in time during online activities associated with each of the four laboratory exercises are shown in Figures 2, 3, and 4. Each figure caption lists the question that was asked of the students when that particular part of the video, interactive activity, or virtual lab had been reached.

**TABLE 1:** A summary of the four laboratories developed using materials from the Science of Agriculture (SoA), Understanding Western Soils (UWS), and Virtual Labs (VL) websites

Laboratory title	Resources utilized	Website
Understanding Data and Chemistry	Multidimensional Thinking (video)	SoA
	Finding Your Place on the Scale (video)	SoA
	Scientific Graph Reading (interactive activity)	SoA
	Logarithm Calculator (interactive activity)	SoA
	Everything Is Chemical (video)	SoA
	The pH Scale and Meter Calibration (virtual laboratory)	VL
	Testing and Adjusting pH (virtual laboratory)	VL
Soil Chemistry	Cation Exchange (video)	SoA
	Sorption in Everyday Life (video)	SoA
	Sorption: A Close‐Up View (video)	SoA
	Sorption! (interactive activity/virtual laboratory)	SoA
Dryland Soils and Irrigation	Understanding the Sodium Adsorption Ratio (SAR) (video)	UWS
	Unavailability of Water in Saline Soils (video)	UWS
	Dispersion and Flocculation (video)	UWS
	Gypsum and Sodic Soils (video)	UWS
	Runoff and Infiltration (video)	UWS
	Ground Cover (video)	UWS
	Water Sampling (interactive activity/virtual laboratory)	SoA
	Irrigation Training Modules–Overview: Water Treatment System (video)	SoA
	Irrigation Training Modules–Interactive: Test Strip Lab (interactive activity)	SoA
Microbiology and Nitrogen	Nutritious Nitrogen–Fertilizing Chile (interactive activity)	SoA
	Nitrogen and Agriculture (interactive activity)	SoA
	Testing for Corn Mold (virtual laboratory)	VL
	Bacteria Sampling (virtual laboratory)	VL
	Gram Staining (virtual laboratory)	VL
	Using the Microscope (virtual laboratory)	VL

## 4 DISCUSSION

Several studies have shown that well‐developed online activities can benefit student learning (Brevik, 2020; Chang & Wang, 2009; Mayer & Moreno, 2002; Vasiliadou, 2020). Hegerfeld‐Baker (2013) found that the virtual laboratories available on the VL website increased interest in agricultural science careers among students who did not have access to traditional laboratory science in high school. The design process used to create many of the SoA, UWS, and VL activities has also been shown to be highly effective (Chamberlin et al., 2012; Foster, 2008; Mayo, 2007 ). Furthermore, specific activities within the SoA website have been evaluated and demonstrated to enhance student learning (Ulery et al., 2020). Some concepts such as CEC or sorption are more easily visualized using computer graphics and online tools (Ulery et al., 2020). It is important to note that no data were collected regarding student responses to this approach, as this was an exercise in surviving spring 2020 rather than a thought‐out and designed study. Therefore, this is a case study to share the approach used, the reasons for it, the personal lessons learned, and thoughts for the future with others who teach similar classes. That being said, because of previous work looking at the use of resources such as these to enhance student learning, there was a high degree of confidence that utilizing the activities available on the SoA, UWS, and VL websites would provide a meaningful learning experience to the DSU SOIL 210 students.

The SoA website worked well for SOIL 210, in part because of the timing; there were many activities available that corresponded to topics being covered in lecture during the second half of the semester. The majority of the activities available on SoA and its related websites were utilized in creating the four online laboratories (13 of 17 activities on SoA, 6 of 15 activities on UWS, and 6 of 8 activities on VL; in all, 25 of the 40 activities that were available in the spring of 2020). In cases where activities were not used, the reasons were:

1. The material had already been covered in SOIL 210 by that point in the semester (e.g., the Particle Size video on the UWS site and the Soil Infiltration: Soil Porosity Testing animation on the SoA site)
2. The material was very similar to something else already being utilized (e.g., the Runoff and Infiltration and Surface Cover and Runoff videos on UWS were very similar to the Ground Cover video that was used; the Magic of Reading Graphs interactive activity on SoA was very similar to the Scientific Graph Reading interactive activity that was used)
3. The material was not seen as being particularly relevant to the SOIL 210 class (the Understanding Water Activity and Controlling Water Activity in Food virtual laboratories on VL; these were developed specifically for food scientists).

Due to the sudden switch in delivery format, not all of the skills that had originally been planned to be covered in the SOIL 210 laboratory after spring break 2020 ended up being covered. In particular, the measurement of certain soil physical properties such as bulk density and using the universal soil loss equation to estimate soil loss were not done. In addition, skills that were covered by the SoA, UWS, and VL websites, such as determining pH or observing selected soil physical processes, such as flocculation, were covered in a manner that allowed the students to see the activity being done or observe the process but did not require that they actually be involved in it. The SoA activities have been shown to enhance student learning of complex soil science concepts when used to supplement classroom activities (Ulery et al., 2020), but to date the use of SoA and its related sites to replace laboratory activities has not been evaluated. Therefore, it is difficult to access how well the original learning outcomes for the SOIL 210 laboratory were addressed by the change in format.

Despite the uncertainties, there are a wide range of topics that are appropriate for an introductory soil science laboratory, and the SoA, UWS, and VL websites provided options that aligned well with topics that were covered in lecture during the final weeks of the semester (Figure 1). These websites provided resources with a strong potential to enhance student learning, which is one of the fundamental goals of laboratory activities. Utilizing the SoA, UWS, and VL websites to get through the last few laboratories in SOIL 210 also stimulated thoughts on the part of the instructor regarding how these online resources could be used to improve SOIL 210 in the future. Specifically, many of the online laboratories built by the instructor to “survive” spring 2020 could be converted into online homework assignments using the SoA, UWS, and VL resources and become a regular part of the learning strategies used in SOIL 210. Based on the findings of Ulery et al. (2020), this should enhance student learning in the class. Doing this would also expand use of the resources available on SoA and UWS to include some of the activities that were not used in spring 2020. Utilizing these activities as part of face‐to‐face laboratories, such as using a video or an interactive activity to introduce a topic, could also enhance student learning. Other ideas for the virtual learning tools on these sites include using them as extra credit to encourage students to spend more time learning important concepts or as make‐up assignments for students who miss face‐to‐face class sessions.

Future needs include development of more online activities. As discussed above, many of the activities currently available correspond well to the second half of the SOIL 210 semester, but there are fewer activities that align with topics taught during the first half of the semester, at least as the class is taught at DSU. These earlier topics include soil formation, soil classification, soil physical properties, soil water, and soil aeration and temperature. The SoA website has activities that focus on soil water movement and storage and water infiltration, and UWS has videos on particle size and infiltration. However, activities that focus on soil formation, soil classification, and soil aeration and temperature are currently lacking. Good additions to the currently available resources would include a video on the formation of soil horizons, interactive activities involving soil mineralogy models, and demonstrations of how heat moves through the soil. The NMSU team is open to feedback and collaborations on topics suited for videos, virtual labs, demonstrations, and so forth, so the SoA and UWS resources can be improved to enhance soil science education. New materials are also currently in development and will be added to SoA soon, so users are encouraged to check back periodically to see if new activities that address items of interest have been added. A final future need is further studies investigating the effectiveness of these online activities as learning tools for students in various environments (e.g., as virtual laboratories, online homework assignments, or pre‐labs). Preliminary research suggests that students showed more proficiency with hands‐on lab activities if they first complete a related VL as a pre‐lab exercise (Peterson, 2014).

## 5 CONCLUSIONS

Spring 2020 presented many challenges in the transition to distance delivery, but the resources freely available through the SoA, UWS, and VL websites helped allow learning to continue. Studies have shown animations, interactive activities, and virtual labs such as those found at the SoA, UWS, and VL websites to be effective ways to engage students and reinforce difficult‐to‐grasp soil science concepts, but more research is needed on the effectiveness of the animations and interactive activities at providing a virtual laboratory experience and how student learning compares with the learning achieved in a traditional laboratory setting. Future needs include developing new activities to address aspects of soil science that are not currently covered and evaluating the effectiveness of these learning options in formats not yet studied.

In the opinion of the senior author, the SoA, UWS, and VL websites provided a better laboratory experience for the SOIL 210 students than some other alternatives that were considered, such as assigning the students worksheets with problems to answer or videos to watch. The materials on the websites provided better images than most worksheets and those images are animated, rather than still. The websites are interactive, whereas videos are passive. And, the website animations walked the students step‐by‐step through several laboratory and field procedures that are important in soil science. From this perspective, the SoA, UWS, and VL materials provided a satisfying option to complete the SOIL 210 laboratories.

The online materials were not, again in the senior author's opinion, a complete replacement for a face‐to‐face laboratory. Laboratories are a way to provide students with hands‐on opportunities to learn procedures that are important in a discipline. As an example, the ability to click a mouse and get a virtual pipette to transfer a given amount of chemical from one virtual beaker to another does not mean the student could perform that same operation with a physical pipette in a laboratory, which is a skill they might develop in a face‐to‐face lab exercise. Therefore, although the SoA, UWS, and VL websites provided a meaningful way to continue the SOIL 210 laboratory in a mandated distance delivery environment, it would be the senior author's preference to conduct laboratories face‐to‐face whenever possible.

The need to move to distance delivery in spring 2020 also led to serious consideration about how the SoA, UWS, and VL websites could be used in SOIL 210 in the future to enhance student learning. As we move into a “new normal” situation in higher education, changes made to survive the spring 2020 semester will alter how SOIL 210 is taught, most likely with the development of online homework assignments that revolve around the SoA, UWS, and VL websites. In many cases the same questions that were used for laboratories in spring 2020 could be utilized in homework assignments in the future. In this sense the COVID‐19 inspired distance delivery mandate will likely improve the SOIL 210 class going forward.

## CONFLICT OF INTEREST

The authors declare no conflicts of interest.
